# Forkhead transcription factor FoxF1 interacts with Fanconi anemia protein complexes to promote DNA damage response

**DOI:** 10.18632/oncotarget.6422

**Published:** 2015-11-28

**Authors:** Arun Pradhan, Vladimir Ustiyan, Yufang Zhang, Tanya V. Kalin, Vladimir V. Kalinichenko

**Affiliations:** ^1^ Division of Pulmonary Biology, Perinatal Institute of Cincinnati Children's Research Foundation, Cincinnati, OH 45229, USA

**Keywords:** FoxF1 transcription factor, Fanconi anemia protein complex, DNA repair, tumor cells

## Abstract

Forkhead box F1 (Foxf1) transcription factor is an important regulator of embryonic development but its role in tumor cells remains incompletely understood. While 16 proteins were characterized in Fanconi anemia (FA) core complex, its interactions with cellular transcriptional machinery remain poorly characterized. Here, we identified FoxF1 protein as a novel interacting partner of the FA complex proteins. Using multiple human and mouse tumor cell lines and *Foxf1*^+/−^ mice we demonstrated that FoxF1 physically binds to and increases stability of FA proteins. FoxF1 co-localizes with FANCD2 in DNA repair foci in cultured cells and tumor tissues obtained from cisplatin-treated mice. In response to DNA damage, FoxF1-deficient tumor cells showed significantly reduced FANCD2 monoubiquitination and FANCM phosphorylation, resulting in impaired formation of DNA repair foci. FoxF1 knockdown caused chromosomal instability, nuclear abnormalities, and increased tumor cell death in response to DNA-damaging agents. Overexpression of FoxF1 in DNA-damaged cells improved stability of FA proteins, decreased chromosomal and nuclear aberrations, restored formation of DNA repair foci and prevented cell death after DNA damage. These findings demonstrate that FoxF1 is a key component of FA complexes and a critical mediator of DNA damage response in tumor cells.

## INTRODUCTION

Cancer cells possess diverse phenotypic features which distinguish them from their nonmalignant counterparts. The most prominent features of cancer cells are enhanced cell proliferation and reduced apoptotic rates along with an inherent capability to invade and metastasize [1–2]. Malignant cells also possess chromosomal instability which is a crucial feature needed for the tumor progression [3]. In order to attain a state of chromosomal instability, cancer cells inhibit DNA damage response by accumulating inactivating mutations in genes critical for DNA repair pathways, including genes encoding subunits of Fanconi anemia complex [4]. Fanconi anemia (FA) is a rare pediatric chromosome instability disorder characterized by developmental defects, bone marrow failure and higher susceptibility to both hematologic and nonhematologic cancers [4–8]. Other symptoms of FA include endocrine and gastrointestinal abnormalities, limb deformities and skin hyperpigmentation [6–8]. The higher cancer susceptibility of FA patients is due to defects in the DNA damage repair pathway. The hallmark cellular feature of cells derived from FA patients includes chromosomal instability and hypersensitivity to crosslinking agents, such as mitomycin C (MMC), Cisplatin and diepoxybutane (DEB) [9–10]. This rare cancer-prone disease has generated wide-spread attention because the proteins involved in FA have led to the elucidation of a repair pathway for interstrand DNA crosslinks (ICL).

To date, sixteen FA tumor suppressor genes that act together to protect cells against the stress generated by ICL agents or endogenous metabolites have been identified [8, 11, 12]. The proteins encoded by these genes form multiple complexes to orchestrate ICL repair. The Fanconi anemia defect results from bi-allelic mutation of any one of sixteen known FA genes. The FA core complex, comprised of 8 proteins (FANCA, -B, -C, -E, -F, -G, -L and -M), along with 6 associated factors (FAAP20, FAAP100, FAAP24, HES1, MHF1 and MHF2), acts as an E3 ligase to ubiquitinate the FANCI/FANCD2 (I/D2) complex, resulting in activation of a downstream DNA repair response [7, 10, 12–14]. In addition to the FA core and I/D2 complex, FANCD1/BRCA2, FANCN/PALB2, FANCJ/BACH1, FANCO/RAD51C, FANCP/SLX4 and the associated proteins RAD51, RAD18 and FAN1 participate in FA DNA repair response. All these proteins function together to facilitate DNA interstrand cross-link and other DNA damage response repairs by recovering and stabilizing stalled replication forks. The deubiquitinating (DUB) enzyme USP1 (ubiquitin-specific peptidase 1) along with UAF1 (USP1-associated factor 1), deubiquitinates the FANCD2-I complex to complete the repair process [12, 13].

Recent studies demonstrated that FA proteins participate in cellular processes well beyond DNA repair [12, 15]. FANCD2 protein has been shown to promote nucleosome assembly, and it also interacts directly with the chromatin-remodeling enzyme Tip60 [16, 17]. FANCD2 protein has also been reported to act as a transcriptional activator of tumor suppressor gene Tap63 [18]. FA proteins have also been implicated in mitosis and cytokinesis. FANCI and FANCD2 proteins have been shown to localize to ultrafine DNA bridges linking sister chromatids during cell division [19]. FA proteins interact with Hes1, Runx1 and Runx3 transcription factors that regulate stability of FA complexes [20–21]. Despite the identification and characterization of multiple proteins in the FA protein complexes, their interactions with cellular transcriptional machinery remain poorly characterized.

Forkhead Box F1 (FoxF1) transcription factor is a critical mediator of lung development and lung injury/ repair [22–27]. Heterozygous deletions and point mutations in the *FOXF1* gene locus were found in patients with Alveolar Capillary Dysplasia with Misalignment of Pulmonary Veins (ACD/MPV), a rare congenital disorder characterized by severe defects in development of the alveolar capillary network. Global deletion of *Foxf1* in mice (*Foxf1* ^−/−^) is embryonic lethal [28], and FoxF1 heterozygous mice (*Foxf1* ^+/−^) exhibit alveolar capillary dysplasia and abnormal lung repair [23, 29, 24]. During lung development, the Sonic hedgehog signaling pathway stimulates expression of FoxF1 [29], which in turn, transcriptionally activates expression of VEGF receptors and promotes VEGF signaling in embryonic endothelial cells [22]. FoxF1 binds to serum response factor (SRF) and myocardin to regulate SRF signaling in smooth muscle cells [30, 31]. Recent studies reported deregulation of *FOXF1* gene in human cancers [32]. FoxF1 was identified as a target gene of tumor suppressor p53, forming a transcriptional network which regulates cancer cell migration and invasiveness [33]. Genomic deletions in *FOXF1* gene locus have been found in prostate cancer samples [33–34], whereas epigenetic inactivation of *FOXF1* promoter has been reported for breast invasive ductal carcinomas [35]. In contrast, FoxF1 expression was increased in patched-associated tumors, such as basal cell carcinoma, medulloblastoma and rhabdomyosarcoma [36–38], underlying the importance of FoxF1 in aberrant Hedgehog signaling in human cancers. Overexpression of FoxF1 promoted invasion and metastasis of breast carcinomas [39] and enhanced the tumor-promoting properties of cancer-associated fibroblasts [40].

In the current study, we demonstrated that FoxF1 is a novel interacting partner for the FA protein complexes. FoxF1 physically binds to the FA core and I/D2 complexes, induces their binding to chromatin, promotes DNA repair and protects tumor cells from cell death in response to DNA-damaging agents. Our findings indicate that FoxF1 protein is a key regulatory component of the FA pathway and a critical mediator of DNA damage response.

## RESULTS

### FoxF1 transcription factor physically interacts with FA proteins complexes

**Figure 1:**
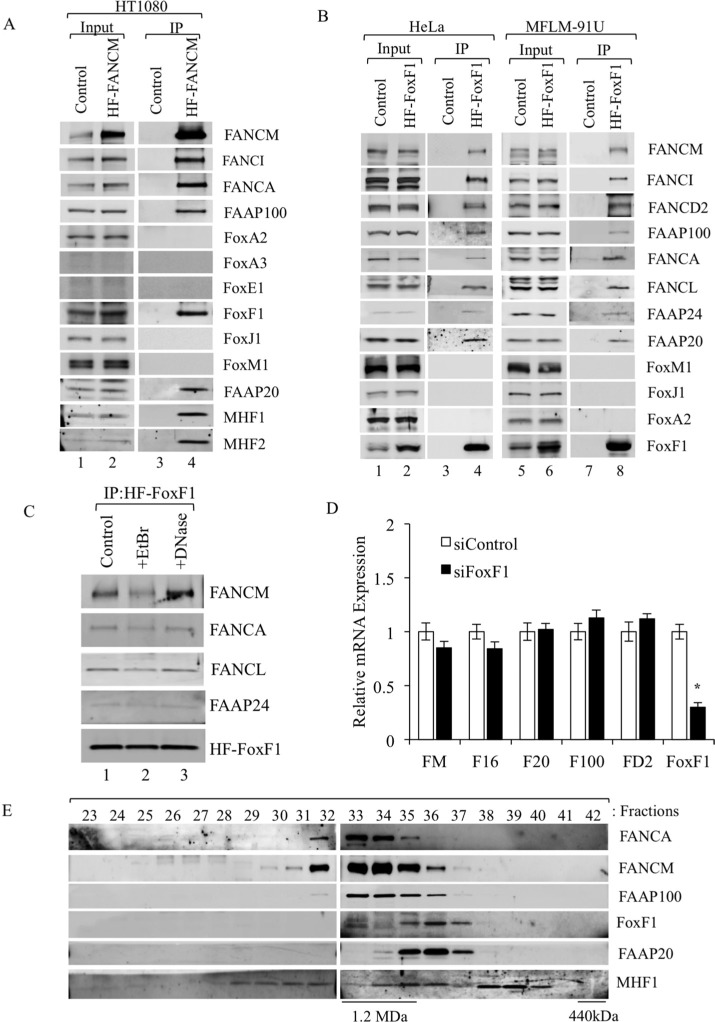
FoxF1 interacts with FA complex proteins (**A**) Immunoblots show co-immunoprecipitation of endogenous FoxF1 protein with HF-FANCM and other proteins of the FA core complex in HT1080 cells. FoxA2, FoxJ1 and FoxM1 did not interact with HF-FANCM. FoxA3 and FoxE1 were not detected in HT1080 cells. (**B**) Immunoblots show the presence of FA proteins in IP fractions after HF-FoxF1 purification. Nuclear extracts of HeLa or MFLM-91U cells stably expressing HF-FoxF1 were subjected to 2-step affinity chromatography using anti-Flag and nickel affinity columns. The eluates were analyzed by western blotting using antibodies for the indicated proteins. Cells transduced with vector alone were used as controls. (**C**) Ethidium bromide and DNase do not influence interactions of HF-FoxF1 with FANCM, FANCA, FANCL and FAAP24. HeLa cells stably expressing HF-FoxF1 were used for IP. (**D**) qRT-PCR was used to examine expression of indicated FA genes in FoxF1-depleted cells. Depletion of FoxF1 from MFLM-91U cells was performed by siRNA transfection. Total RNA was isolated and examined by qRT–PCR. Expression levels were normalized to β-actin mRNA. A *p*-value < 0.05 is shown with asterisk (*). FANCM (FM), FAAP16 (F16), FAAP20 (F20), FAAP100 (F100) and FANCD2 (FD2). (**E**) Immunoblot shows overlapping gel filtration profiles and co-fractionation of FoxF1 with FA proteins. Superose 6 gel filtration columns were used for co-fractionation.

Since protein-protein interactions between FA complexes and cellular transcriptional machinery are not well understood, we used 2-step affinity purification coupled with immunoblotting to screen for transcription factors physically bound to FANCM protein, an FA core complex component [41, 42]. HT1080 tumor cells stably expressing His-Flag tagged FANCM (HF-FANCM) protein [41] were used for co-immunoprecipitation (co-IP). Among multiple transcription factors screened in this assay, endogenous FoxF1 protein was found to bind the HF-FANCM along with its known interacting partners, such as FANCA, FANCI, FAAP100, FAAP20, MHF1 and MHF2 (Figure 1A). To demonstrate specificity of FoxF1 /FA protein interactions, we also tested for the presence of other forkhead box transcription factors in FA core complex, such as FoxA2, FoxA3, FoxE1, FoxJ1 and FoxM1. While HT1080 cells expressed only FoxA2, FoxM1 and FoxJ1, none of them was found to be interacting with FANCM (Figure 1A). In order to confirm the interaction of FoxF1 with FA complex proteins, reciprocal co-IP experiments were performed. Using retroviral-mediated gene transfer, we generated two distinct cell lines stably expressing FoxF1 protein, which contains an N-terminal Flag and a C-terminal (His)6-tag (HF-FoxF1). HF-FoxF1 and its interacting proteins were purified from nuclear extracts by a 2-step affinity purification approach. Immunoblot analysis of proteins in the FoxF1 purified fraction showed interaction with multiple FA core complex proteins, including FANCM, FAAP100, FANCA, FANCL, FAAP24 and FAAP20 (Figure 1B). FoxF1 also bound to FANCI and FANCD2 proteins that are main components of downstream I/D2 FA complex (Figure 1B). Other Fox proteins, such as FoxM1, FoxJ1 and FoxA2 did not interact with FA complexes (Figure 1B), confirming a specificity of FoxF1/FA protein interactions. Interactions between the FoxF1 and FA complex proteins were not due to DNA contamination of the protein lysate, because neither ethidium bromide nor DNase, which precipitate and degrade DNA, respectively, prevented FoxF1/FA protein interactions (Figure 1C). To determine whether endogenous FoxF1 co-fractionates with FA complex proteins, we performed a superpose-6 gel filtration experiment of nuclear extract from HeLa cells. The gel filtration profile of endogenous FoxF1 protein overlapped with that of several FA core proteins, such as FANCA, FANCM, FAAP100, FAAP20 and MHF1 (Figure 1E), confirming that FoxF1 interacts with the FA proteins. Altogether, these experiments demonstrate that FoxF1 specifically binds to the FA core and I/D2 FA complexes through protein-protein interactions.

### FoxF1 increases binding of FA complex to chromatin

**Figure 2:**
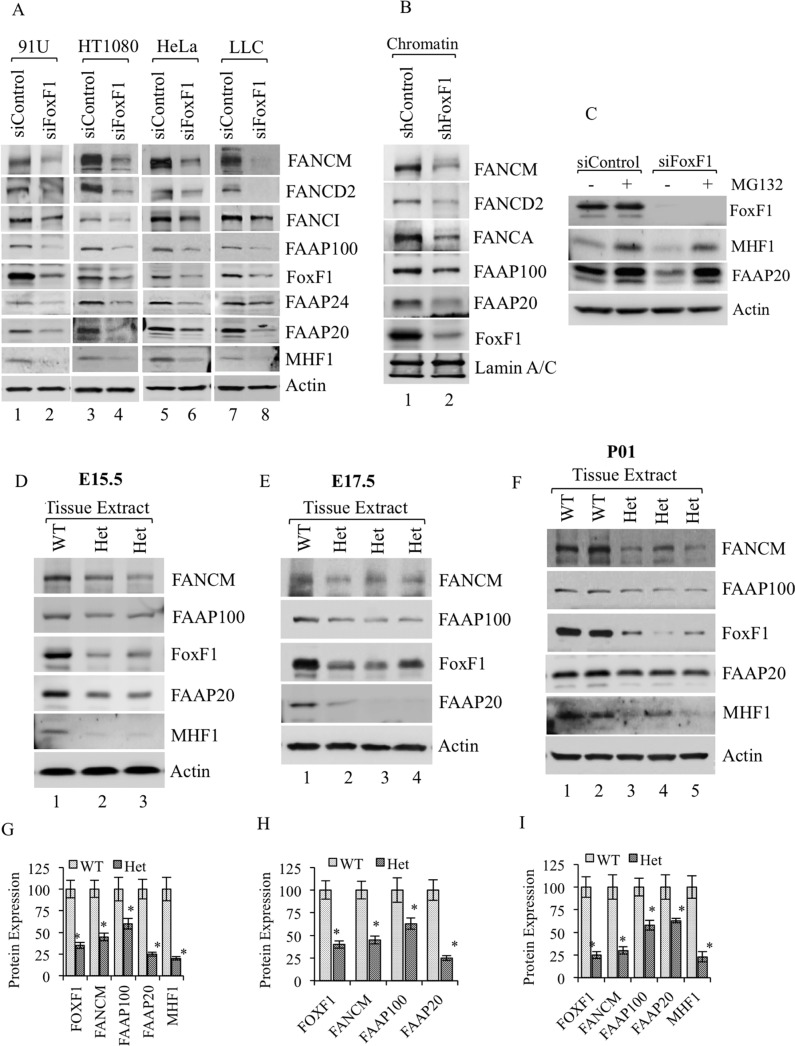
FoxF1 regulates stability of FA complex proteins (**A**) Immunoblots show FA proteins in different cell lines transfected with either control or FoxF1 specific siRNA. Depletion of FoxF1 reduced the levels of FA proteins in whole cell lysates. β-actin serves as loading control. (**B**) Immunoblots showing the association of FANCM, FANCD2, FANCA, FAAP100, FAAP20 and FoxF1 proteins with chromatin in HUVEC cells transfected with either shControl or shFoxF1–3- UTR. Lamin A/C serves as a loading control for the chromatin fraction. (**C**) Immunoblot shows FoxF1, MHF1 and FAAP20 protein levels in total cell lysates of siControl and siFoxF1 treated cells that were cultured in the presence or absence of MG132. (**D–F**) Immunoblots show reduced levels of FoxF1 and FA proteins in Foxf1^+/−^ mice as compared to wild type (WT) littermates. Total lysate was prepared from lungs of E15.5, E17.5 embryos or newborn mice (P01). β-actin serves as loading control. (**G–I**) Graph shows densitometric quantification of western blots presented in (D–F). Protein levels were normalized to β-actin. A *p*-value < 0.05 is shown with asterisk (*).

The absence of one interacting protein has been shown to decrease the stability of the entire FA and I/D2 complexes [7, 41]. Consistent with these studies, siRNA-mediated knockdown of FoxF1 in four different cell lines reduced steady-state levels of FANCM, FANCD2, FANCI, FAAP100, FAAP24, FAAP20 and MHF1 proteins as shown by Western blot (Figure 2A). To rule out the possibility that FoxF1 transcriptionally regulates the expression of FA genes, qRT-PCR was used to examine FA mRNAs in FoxF1-depleted cells. Depletion of FoxF1 did not influence mRNA levels of FA genes (Figure 1D). Analysis of chromatin-associated proteins further revealed that depletion of FoxF1 reduced FA protein levels in the chromatin-bound nuclear fraction (Figure 2B), suggesting that FoxF1 promotes association of the FA core and I/D2 complexes with the chromatin. Treatment with proteasome inhibitor MG132 resulted in recovery of MHF1 and FAAP20 protein levels even in FoxF1-depleted cells (Figure 2C), indicating that FoxF1 regulates degradation of the FA proteins.

Since *Foxf1* ^−/−^ mouse embryos die very early in development, we used heterozygous *Foxf1* ^+/−^ embryos to examine FA protein levels. Reduced levels of the FA complex proteins FANCM, FAAP100, FAAP20 and MHF1 were found in lung tissue obtained from E15.5 and E17.5 *Foxf1* ^+/−^ embryos that contained diminished FoxF1 protein levels as shown by Western blot (Figure 2D–2E and 2G–2H). Similar data were obtained from lung tissue of *Foxf1*^+/−^ newborn mice (Figure 2F and 2I). Thus, inactivation of FoxF1 *in vitro* and *in vivo* reduces FA protein levels.

### DNA damage increases association of FoxF1 with FA proteins

**Figure 3:**
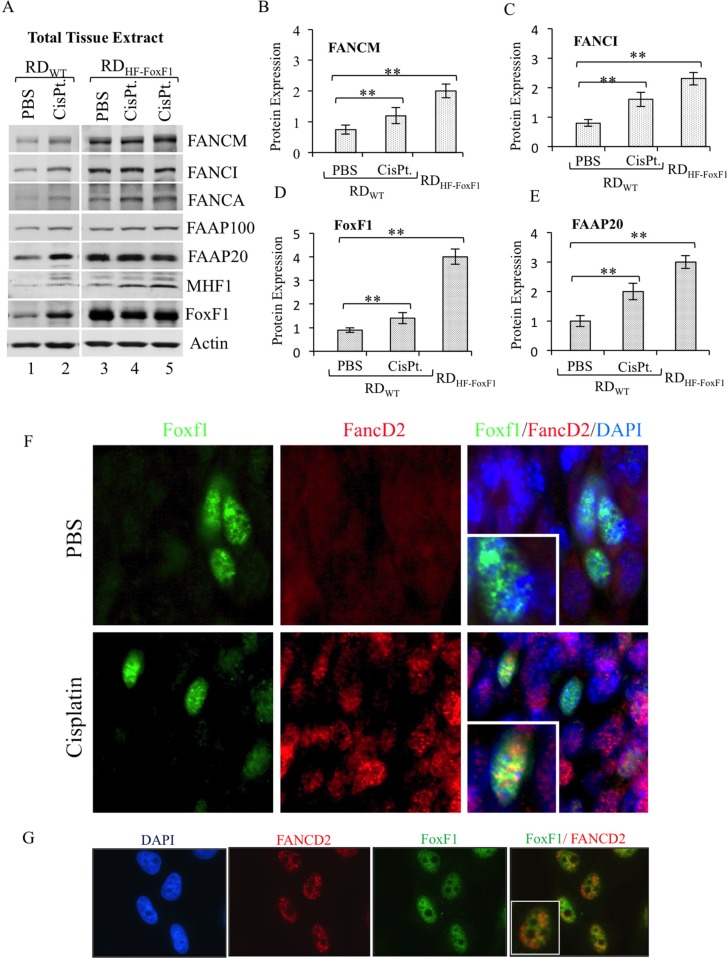
Overexpression of FoxF1 increases the levels of FA proteins in tumor tissue (**A**) Immunoblots show the levels of endogenous FoxF1 and FA proteins in mouse rhabdomyosarcoma tumor tissues. Tumors were harvested from mice inoculated with rhabdomyosarcoma cells stably expressing either HF-FoxF1 (RDHF-FoxF1) or control vector (RDWT). Tumor-bearing mice were treated with cisplatin 24 hr prior to the tumor harvest. β-actin serves as loading control. (**B–E**) Bar graph shows the densitometric quantification of western blots shown in (A). A *p*-value < 0.05 is shown with asterisk (*). (**F**) Representative immunostaining shows colocalization of FoxF1 and FANCD2 (arrows) in a subset of cells located in cisplatin-treated tumor. Sections from HF-FoxF1 rhabdomyosarcoma tumors were stained for FoxF1 (Green) and FANCD2 (Red). DAPI (blue) was used to stain nuclei. (**G**) Co-localization of FoxF1 and FANCD2 was observed in HU-treated HeLa cells. HeLa cells were exposed to HU for 16 h.

Previous studies have shown that stability of FA complex proteins is increased after DNA damage [7, 10, 41]. Therefore we used cisplatin, a known DNA-damaging agent, to examine FA protein levels in FoxF1-expressing tumor cells *in vivo*. Mice bearing FoxF1-overexpressing or control rhabdomyosarcoma tumors were treated with cisplatin to induce DNA damage response. Cisplatin treatment increased FoxF1 protein levels as shown by Western blot of tumor lysate (Figure 3A; lanes 1 and 2). Increased FoxF1 levels in cisplatin-treated tumors were associated with an increase in FA complex proteins, including FANCM, FANCI, FAAP20, FAAP100 and MHF1 (Figure 3A). Interestingly, stable overexpression of FoxF1 in rhabdomyosarcoma cells was sufficient to increase FA protein levels in tumor tissue even in the absence of DNA damage (Figure 3A, lanes 3–5). Thus, FoxF1 increases the FA protein levels in tumor tissue in a mouse model of rhabdomyosarcoma.

Since depletion of FoxF1 reduced the association of the FA core and I/D2 complexes with chromatin (Figure 2B), we examined whether FoxF1 is physically present in DNA repair foci. FANCD2, a marker of DNA repair foci [16, 43, 44] co-localized with FoxF1 in nuclei of tumor cells treated with hydroxyurea, which induces a DNA-damage response (Figure 3G). Co-localization of FoxF1 with FANCD2 was also observed in a subset of tumor cells located within cisplatin-treated rhabdomyosarcoma tumors (Figure 3F). Thus, FoxF1 is physically present in DNA repair foci induced by DNA damaging agents.

**Figure 4:**
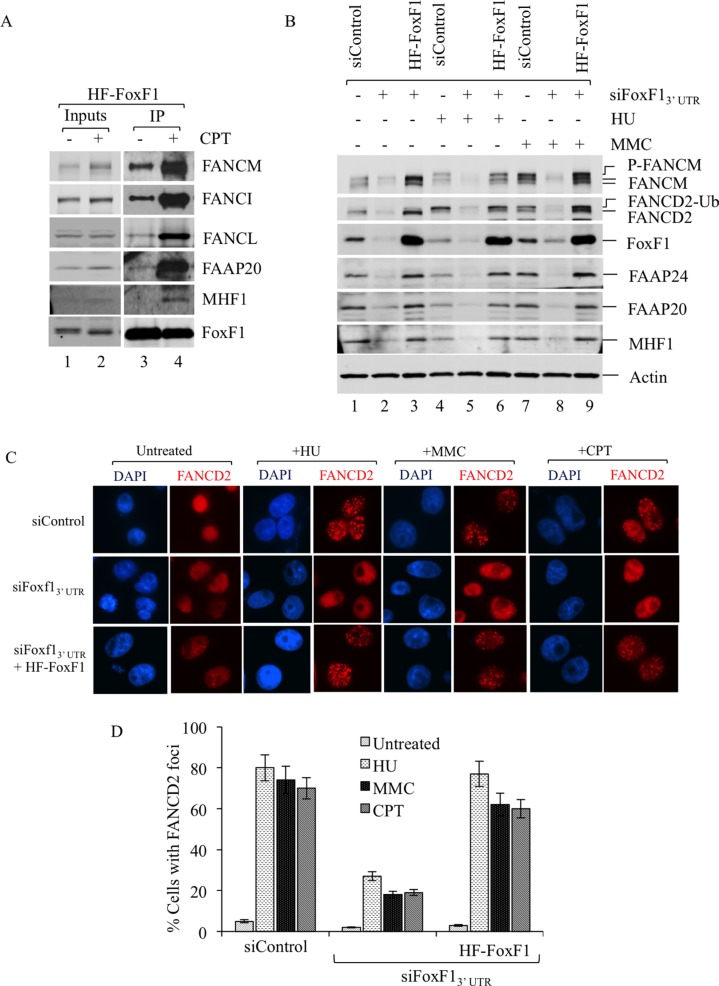
FoxF1 is required for the activation of the FA pathway (**A**) Immunoblots show increased association of the FA complex proteins with FoxF1 in response to DNA damage. HeLa cells stably expressing HF-FoxF1 were treated with Camptothecin or vehicle for 16 h. HF-FoxF1 was purified from nuclear extract with anti-Flag M2 agarose followed by metal affinity resin. (**B**) Immunoblots show that inhibition of FoxF1 by siRNA reduces FANCD2 monoubiquitination and FANCM phosphorylation. After treatment with either mitomycin (**C**) (MMC) or hydroxyurea (HU) for 16 hr, FoxF1 and FA proteins were detected by Western blot. The monoubiquitanted isoform of FANCD2 (FANCD2-Ub) and phosphorylated isoforms of FANCM (P-FANCM) are indicated. β-actin was used as a loading control. (C) FoxF1-depletion impairs formation of FANCD2 positive foci after DNA damage. Forty-eight hours after siRNA transfection HeLa cells were treated with HU (1.5 mM), MMC (100 ng/mL) or Camptothecin (20 nM) for 16 hr. Slides were stained for FANCD2 (red) and counterstained with DAPI. Knockdown of FoxF1 by siFoxF1–3′ UTR reduced formation of FANCD2 foci. Expression of HF-FoxF1 rescues ability of DNA-damaged cells to form foci. (**D**) Histogram shows quantification of FANCD2 immunostaining. The percentage of cells with 5 or more FANCD2-positive foci was determined by examining at least 150 cells. Histogram shows mean ± SD from 3 independent experiments.

Next, we determined whether FoxF1/FA protein-protein interactions are altered in response to DNA damage. HeLa tumor cells stably expressing HF-FoxF1 were treated with Camptothecin to induce DNA damage response and the binding of FoxF1 with FA proteins was examined by IP. Interaction of HF-FoxF1 with the FA complexes was increased after DNA damage as shown by increased binding of FoxF1 to FANCM, FANCI, FANCL, FAAP20 and MHF1 proteins (Figure 4A). Thus, DNA damage increases association of FoxF1 with FA complex proteins.

### FoxF1 increases activation of the FA complex in response to DNA damage

Next, we examined downstream events in the FA DNA repair pathway in the presence or absence of FoxF1. Specifically, siRNA-mediated depletion of FoxF1 was used to determine whether FANCD2 mono-ubiquitination and FANCM phosphorylation, critical steps in activation of the FA DNA repair complex [16, 41, 43, 45], are regulated by FoxF1. In response to DNA damage mediated by HU and MMC, both FANCD2 mono-ubiquitination and FANCM phosphorylation were detected in tumor cells transfected with control siRNA (Figure 4B, lanes 4, 7). FoxF1 knockdown decreased protein levels of FANCM, FANCD2, FAAP24, FAAP20 and MHF1 (Figure 4B). In addition, FANCM phosphorylation did not occur and FANCD2 mono-ubiquitination was significantly reduced in tumor cells transfected with FoxF1-specific siRNA (Figure 4B, lanes 5, 8). Thus, depletion of FoxF1 decreased FA protein stability and prevented activation of the FA complex in response to DNA damage. To determine whether this effect is a direct consequence of FoxF1 knockdown, but not an off-target effect of siRNA-transfection, we restored FoxF1 expression in FoxF1-depleted cells using the exogenous HF-FoxF1 construct, which is resistant to siFoxF1 targeting. HF-FoxF1 overexpression rescued FANCD2 mono-ubiquitination and FANCM phosphorylation in response to DNA damage (Figure 4B, lanes 6 and 9). In addition, HF-FoxF1 overexpression improved stability of FA core complex and its associated sub-complexes in FoxF1-depleted tumor cells (Figure 4B). Thus, FoxF1 promotes activation of FA complex after DNA damage.

### FoxF1 is required for formation of DNA repair foci in response to DNA damage

Mono-ubiquitination of FANCD2 protein is known to target FANCD2 to DNA repair foci [43, 44]. Consistent with these studies, HU, MMC or CPT DNA damaging agents induced formation of FANCD2 foci in nuclei of HeLa tumor cells (Figure 4C–4D). SiRNA-mediated depletion of FoxF1 impaired FANCD2 targeting to DNA repair foci (Figure 4C–4D). Expression of exogenous HF-FoxF1 protein in FoxF1-deficient cells rescued the formation of FANCD2 foci in response to DNA damage (Figure 4C–4D). These results demonstrate that FoxF1 induces FANCD2 translocation to DNA repair foci.

### FoxF1 promotes cell survival and genome maintenance after DNA damage

**Figure 5:**
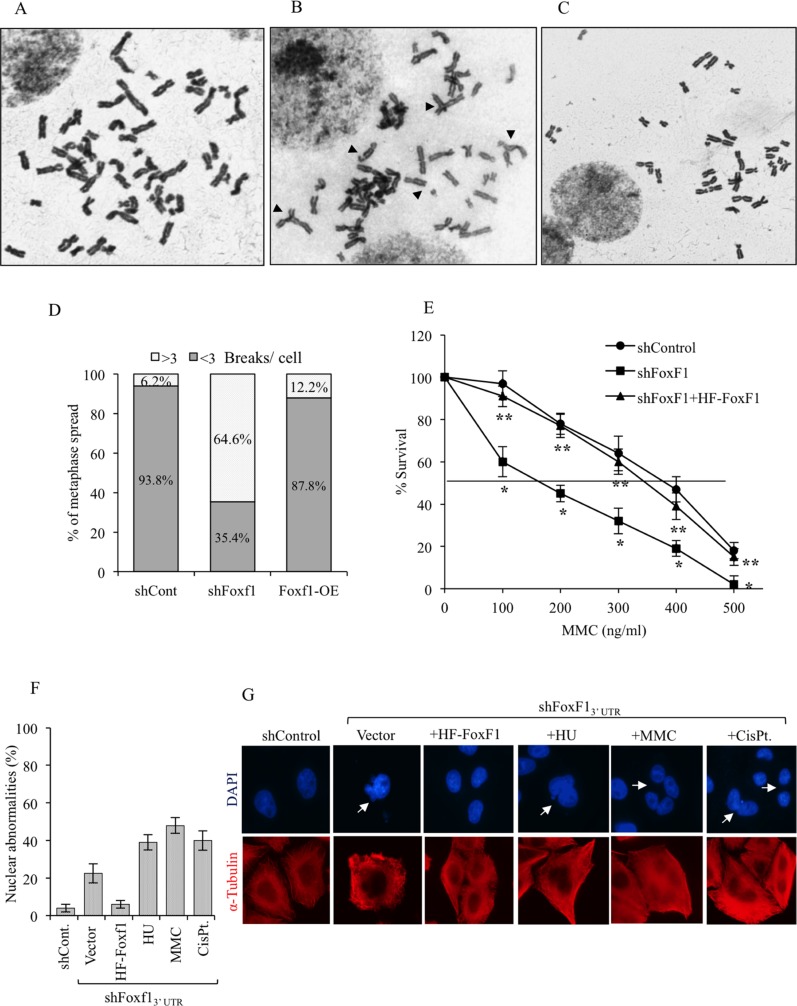
FoxF1 deficiency causes genomic instability after DNA damage (**A–C**) A representative image of metaphase spreads showing chromosomal abnormalities (arrow heads) in FoxF1-depleted cells after DNA damage. Human HEK293 cells stably expressing shControl (A), shFoxF1 (B) or shFoxF1 + HF-FoxF1 (C) were exposed to MMC and then treated with colcemid. (**D**) Quantification of chromosomal aberrations frequencies in MMC-treated cells. Fifty metaphase spreads were examined from two independent experiments. (**E**) Survival curve shows that FoxF1-depletion causes increased sensitivity to MMC. HeLa cells were transduced with either shControl or shFoxF1 and subsequently exposed to different concentrations of MMC. Visible colonies from 200 cells were counted after 10 days. *indicates statistically significant differences (*p* < 0.05) between shFoxF1-transduced cells and cells transduced with control shRNA (shControl). **indicates significant differences (*p* < 0.05) between shFoxF1+HF-FoxF1 and shFoxF1 samples. Each experiment was performed in triplicate. (**F**) Quantification of frequency of nuclear abnormalities after FoxF1 knockdown. Data show the mean percentage of cells exhibiting nuclear abnormalities from 3 independent experiments. (**G**) Representative images of cells stained with DAPI and α-tubulin. Nuclear abnormalities are shown with arrowheads.

Defects in FA DNA repair pathway are associated with various chromosomal and nuclear abnormalities, often resulting in cell death [10, 41, 46]. Therefore, we examined the number of chromosomal aberrations in metaphase of FoxF1-depleted tumor cells exposed to DNA-damaging agent. Similar to FA-deficient cells [7, 10, 41], increased number of chromosomal aberrations was observed in MMC-treated tumor cells deficient for FoxF1 (Figure 5A–5D). Overexpression of HF-FoxF1 decreased the number of chromosomal aberrations in FoxF1-depleted cells (Figure 5A–5D). Furthermore, FoxF1 knockdown increased the frequency of nuclear abnormalities in cultured tumor cells (Figure 5F–5G). This phenotype was also corrected by overexpression of HF-FoxF1 (Figure 5F–5G). Thus, similar to FA proteins FoxF1 regulates genome maintenance after DNA damage.

Since defects in the FA DNA repair pathway are associated with hypersensitivity of tumor cells to DNA crosslinking agents [46–48], we examined functional consequences of FoxF1 depletion on cell survival after DNA damage. Compared to control siRNA, FoxF1-depleted tumor cells showed a dose-dependent reduction in survival in response to MMC (Figure 5E). Cell survival in FoxF1-deficient cells was rescued by overexpression of HF-FoxF1, indicating that FoxF1 protects MMC-treated cells from cell death.

Collectively, our findings indicate that the FA DNA repair pathway is dependent on FoxF1 transcription factor, which directly binds to FA protein complexes, mediating its activation, translocation to DNA repair foci and binding to chromatin.

## DISCUSSION

Previous studies with FoxF1-deficient mice have shown that FoxF1 is an important transcriptional regulator of embryonic development [49–51], however, its role in DNA repair of tumor cells remains uncharacterized. In the present study, we demonstrated that FoxF1 induces DNA repair and directly interact with the FA core and I/D2 complexes. In contrast, other Fox proteins (FoxM1, FoxJ1 and FoxA2) do not bind to FA complexes. The interaction of endogenous FoxF1 with the FA proteins was shown by co-IP experiments, as well as a reciprocal IP's using ectopically expressed FoxF1 protein. Together with a similar superpose-6 gel filtration profile, our results strongly suggest that FoxF1 associates with the FA complex via protein-protein interactions. Interestingly, ethidium bromide but not DNase reduced the association of FoxF1 with FANCM, FANCA and FANCL, suggesting that DNase-resistant DNA can contribute to FoxF1/FA protein interactions.

The expression levels of individual proteins in multi-protein complexes are often coordinated in order to achieve proper assembly and function of that complex. Previous studies of FA complexes suggest interdependence of FA proteins on expression and stability of individual protein subunits [10, 41, 46, 48]. For example, the absence of FANCA made FANCG and FANCL proteins unstable [42]. Stability of FAAP100 protein was decreased in the absence of either FANCB or FANCL [52]. Knockdown of MHF1 resulted in decreased stability of FANCM and MHF2 proteins [41], whereas FANCA and FAAP20 are dependent on each other for their protein stability [7]. In the present study, we observed that depletion of FoxF1 from various cell lines, including MFLM-91U, HeLa, HT1080 and LLC tumor cells, reduced steady-state levels of FA proteins but did not affect their mRNA levels. These results suggest that FoxF1 increases stability of the FA protein complexes. Consistent with this hypothesis, proteasome inhibitor MG132 increased FA protein levels in FoxF1-depleted tumor cells. Therefore, it is possible that FoxF1 prevents proteasome-mediated degradation of the FA proteins.

Interestingly, diminished FA protein levels were found in lung tissue of *Foxf1* ^+/−^ mice and embryos. *Foxf1* ^+/−^ mice exhibit alveolar capillary dysplasia (ACD), which results from abnormal development of pulmonary capillaries [23]. While the role of FA genes in lung development is unknown, patients with FA mutations do not develop ACD. Therefore, it is unlikely that reduced FA protein levels contribute to developmental defects in *Foxf1* ^+/−^ lungs.

Published studies demonstrated that MHF1, FANCM and FAAP20 proteins are enriched in the chromatin fraction [7, 41, 42]. FoxF1 was also found in the chromatin fraction, further supporting the involvement of FoxF1 in the FA DNA repair pathway. Interestingly, depletion of FoxF1 reduced the association of the FA core and associated I/D2 sub-complexes with chromatin, suggesting that FoxF1 promotes binding of the FA complexes to chromatin. Furthermore, we found that the interactions between FoxF1 and the FA complexes were increased in response to DNA damage. Depletion of FoxF1 inhibited DNA repair and caused chromosomal aberrations in tumor cells. Thus, similar to other FA proteins, FoxF1 is an essential structural and functional component of the FA DNA repair complexes. Previous studies demonstrated that after DNA damage the FA proteins form nuclear foci in DNA repair sites [16, 43, 46] and that stability of FA proteins was increased after DNA damage [7, 10, 41, 48]. Consistent with these studies, we found that over-expression of FoxF1 in cultured tumor cells and rabdomyosarcoma mouse tumors increased stability of FA complex proteins. FoxF1 co-localized with FANCD2 in nuclear foci of DNA-damaged tumor cells *in vitro* and *in vivo*, suggesting that FoxF1 plays a role in DNA repair via interaction with FA complex proteins. Since FoxF1 levels are increased in alveolar rabdomyosarcomas, the most aggressive type of human rabdomyosarcoma tumors [53], FoxF1 could be involved in DNA repair of tumor cells, mediating increased resistance of these aggressive tumors to chemotherapy.

FANCD2 mono-ubiquitination and FANCM phosphorylation are required for activation of the FA core complex and its targeting to DNA-damaged sites [43, 44]. Consistent with a key role of FoxF1 in the activation and function of the FA pathway, depletion of FoxF1 decreased FANCD2 mono-ubiquitination and reduced FANCM phosphorylation, a phenotype similar to inactivation of several FA complex proteins [7, 10, 45, 47]. After MMC treatment, mono-ubiquitination of FANCD2 was severely impaired in FANCM- or MHF1-depleted cells [41, 47]. Mono-ubiquitination of FANCD2 protein promoted the targeting of the FA complex to DNA repair foci [16, 43]. Consistent with these studies, we observed an accumulation of FANCD2 in nuclear foci of DNA-damaged tumor cells. Interestingly, depletion of FoxF1 resulted in a loss of FANCD2 in nuclear foci, whereas over-expression of FoxF1 was sufficient to restore foci formation in DNA-damaged cells. These data indicate that FoxF1 promotes activation of the FA complex and its translocation to DNA. Interestingly, previous studies implicated various FA complex proteins, such as FANCM, MHF1, FAAP20 and FAAP24, in tumor cell survival after DNA damage. Depletion of one of these individual FA proteins causes spontaneous DNA damage, nuclear abnormalities, chromosomal aberrations and hypersensitivity of tumor cells to DNA crosslinking agents [7, 10, 41, 44, 46]. Consistent with these studies we found that depletion of FoxF1 increased the number of chromosomal and nuclear aberrations and decreased survival in MMC-treated tumor cells. These results indicate that FoxF1 is a critical mediator of the DNA-damage response in tumor cells.

In summary, our findings demonstrated that FoxF1 physically interacts with FA complexes and promotes the FA DNA repair pathway. FoxF1 stabilizes the FA core and I/D2 complexes and increases their activation and targeting to chromatin. Inactivation of FoxF1 leads to increased genomic instability and reduced survival of DNA-damaged tumor cells. Discovery of pharmacological agents that inactivate or decrease FoxF1 in tumor cells can be beneficial for treatment of cancers resistant to conventional chemotherapy.

## MATERIALS AND METHODS

### Cloning, constructs and retroviruses

Analysis of FoxF1 protein sequences from Mouse, Human, Chimpanzee, Xenopus and Zebrafish showed ∼71–94% homology between these species (Supplementary Figure 1A). The amino acid homology between murine and human FoxF1 was ∼94%. Phylogenetic tree reconstruction using the ClustalW method showed that murine FoxF1 is closest to its human counterpart (Supplementary Figure 1B). Murine FoxF1 cDNA was used for this study to create stably transfected cell lines expressing HF-tagged FoxF1 for purification and further characterization of FoxF1 interacting proteins. The pMIEG3 bicistronic retroviral vector was used for protein expression in mammalian cells [41]. The mammalian expression constructs used during the study were pMIEG3:His6-FLAG-FoxF1 and pMIEG3:His6-FLAG-FANCM. Murine FoxF1 was PCR-amplified and an N-terminal Flag (5′-GGCGGATCCGCCACCATGGACT ACAAAGACGATGACGACAAGGACCCCGCGGCGG CGGGC-3′) and a C-terminal (His)6-tag (5′-GGGCCC TCGAGTCAGTGATGGTGATGGTGGTGCATCACAC ACGGCTTGATGTCTTGG-3′) were introduced by PCR, then were cloned into a pMIEG3 vector to generate pMIEG3-HF-Foxf1. PCR was performed using AccuPrime *Pfx* DNA Polymerase according to manufacturer's protocol (Invitrogen). All PCR products were sequenced. Double-tagged FANCM construct was described previously [42] and was a kind gift from Dr. Ruhikanta Meetei. The retroviral particles were made in Cincinnati Children's Viral Vector Core facility and the generation of stable cell lines was as described previously [41].

### Chemicals

Hydroxyurea (Sigma) was suspended in water to a stock concentration of 1 M. Mitomycin C (Sigma) was dissolved in 50% ethanol to a stock concentration of 500 ng/μL. Puromycin (Sigma) was dissolved at a concentration of 10 mg/ml. MG132 (Sigma) was dissolved in DMSO at a concentration of 20 mM. Camptothecin (Sigma) was dissolved in DMSO to stock concentration of 10 mM. Cisplatin (Calbiochem) was dissolved in DMSO at a concentration of 10 mg/ml.

### Cell cultures, siRNA or shRNA knockdown

HeLa, HT1080, LLC, MFLM-91U and Human embryonic kidney 293T cells were cultured and maintained using standard procedures as described previously [7, 51]. Plasmid and siRNA transfection were performed using Lipofectamine 2000 (Invitrogen). For transient knockdown of FoxF1, we used siRNAs targeting either ORF (Mouse: 5′-GAAAGGAGUUUGUCUUCU C-3′ and Human: 5′-GGAAAUGCCAGGCGCUCAAUU-3′) or siFoxF1–3′UTR (Mouse: 5′-CCAGAUACGUGGAAA GAAUUU-3′ and Human: 5′ —GCAGAAAGGUUAAGG CACUUU— 3′). siRNA against a non-targeting sequence (Dharmacon) was used as control in all experiments. All siRNA oligos were purchased from Dharmacon. For retroviral-mediated stable knockdown of FoxF1, a short hairpin RNA (shRNA) targeting 3′UTR of human FoxF1 was used (5′- AAATGTTAGTGGTGGGTCTGA -3′). Stable cell lines were generated using lentiviruses carrying either pLKO.1:Puro-shControl or pLKO.1:Puro-shFoxf1–3′UTR followed by puromycin selection.

### Antibodies

Antibodies against FANCM, FANCA, FANCL, FAAP24, FoxM1, FoxE1, FoxA3, Actin and Lamin A/C were purchased from Santa Cruz Biotechnology (Supplementary Table 1). Other Abs used in this study were as follows: anti-FAAP100 and anti-FAAP20 (GeneTex, Inc), anti-FANCD2 (Abcam), anti-MHF1 and MHF2 (Aviva Systems Biology, Corp.), anti-FoxJ1 and anti-FoxA2 (Seven Hills Bioreagents), anti-FLAG (Sigma) (Supplementary Table 1). M2-agarose beads were purchased from Sigma. Talon metal affinity resin was obtained from BD.

### Immunoprecipitation (IP)

IP experiments were performed from nuclear extracts by using a two-step affinity chromatography protocol as described previously [41]. Briefly, cells expressing either HF-FoxF1 or HF-FANCM were washed with PBS and collected as a pellet. The nuclear extract was processed directly as described below or pre-incubated with or without ethidium bromide (100 μg/mL) or DNase (10 μ/mL) to check whether the interaction is mediated via DNA. The first purification step included the incubation of nuclear lysates with anti-Flag M2 agarose beads (Sigma) followed by elution with 3xFlag peptide. The second purification step was performed by incubating the 3xFlag peptide eluate with Talon metal affinity resin (BD) in the presence of 3 mM imidazole. Bound proteins were eluted using 2x sample buffer. Purified proteins were resolved on SDS-PAGE gel and analyzed by immunoblotting.

### RNA preparation and quantitative real-time RT-PCR (qRT-PCR)

Depletion of FoxF1 from MFLM-91U cells was performed by using siRNA transfection as described above. At 48 h after transfection, total RNA was prepared from siControl and siFoxF1 cells using RNeasy mini kit (Qiagen) and analyzed by qRT-PCR using the StepOnePlus Real-Time PCR system (Applied Biosystems) as described [55–57]. RNA was amplified with Taqman Gene Expression Master Mix (Applied Biosystems) combined with inventoried Taqman mouse gene expression assays: Foxf1, Mm00487497_m1; FANCM, Mm00626872_m1; FAAP16, Mm00510275; FAAP100, Mm01243172; FANCD2, Mm01184611_m1; FAAP20, Mm01266207_m1. Reactions were analyzed in triplicates and expression levels were normalized to β-actin mRNA.

### Cell fractionation and gel filtration

For cell fractionation, cytoplasmic-nucleoplasmic and chromatin-nuclear matrix proteins were purified as described previously [58]. To study the co-fractionation of FoxF1 and FA proteins, Superose 6 gel filtration analysis was done as described previously [41].

### Mice

Male C57BL/6 mice were inoculated with rhabdomyosarcoma cells (RD-WT or RD-HF-FoxF1). Mice were allowed to develop 10 mm tumors prior to cisplatin treatment. Tumor-bearing mice were I.P. injected with saline (control) or cisplatin (7 mg/kg body weight in saline). The mice were sacrificed and the tumor tissue was harvested 24 h after cisplatin treatment for further analysis. For immunoblotting, tumor tissues were homogenized in lysis buffer supplemented with protease and phosphatase inhibitors, and analyzed by western blot as described previously [59, 31, 60]. Tumor tissues were also used to prepare paraffin sections for staining with FoxF1 (R&D Systems) and FANCD2 (Abcam) antibodies as described [51]. Antibody-antigen complexes were detected using either Alexa Fluor 594 or 488 conjugated secondary antibodies (Invitrogen) followed by counter staining with DAPI (Vector Labs, Burlingame, CA). Fluorescence was detected using a Zeiss Axioplan 2 Imaging Universal Microscope with an Axiocam MRm digital camera (Axiovision Release 4.3) as described [61–64]. *Foxf1* ^+/−^ mice were previously generated by homologous recombination [23, 24]. All animal studies were reviewed and approved by the Animal Care and Use Committee of Cincinnati Children's Research Foundation.

### Immunofluorescence

For immunofluorescence experiments cells were grown on poly-D-lysine coated glass coverslips and treated with DNA damaging agents as described previously [41, 65]. The cells were then fixed with paraformaldehyde and permeabilized with 0.3% Triton X-100. Cells were incubated with primary antibodies against FANCD2 (1:1000; Abcam) and FoxF1 (1:200; R&D systems) followed by Rhodamine B or Alexa Fluor 488 conjugated secondary antibody (1:500). Cells were counterstained with DAPI (Vector Laboratories). Images were obtained using confocal microscopy as described [61, 62, 66, 67].

### MMC survival assay and chromosome aberrations analysis

MMC survival assay was done as described previously [7]. For chromosome aberrations analysis, cells were plated in 10 cm dishes and treated with MMC (100 ng/ml) for 16 h. After treatment, cells were exposed to colcemid (Invitrogen) (100 ng/ml) for 2 h, harvested and swollen using 75 mM KCl, then fixed with methanol: acetic acid (3:1). The cell suspension was dropped onto ice-cold, wet glass slides and air-dried. The cells were then stained with Giemsa solution and examined by microscopy.

### Statistical analysis

ANOVA and Student's *T*-test were used to determine statistical significance. *P* values less than 0.05 were considered significant. Values for all measurements were expressed as the mean ± standard deviation (SD).

## SUPPLEMENTARY MATERIALS FIGURE AND TABLE


