# Supplementary material for: Forkhead transcription factor FoxF1 interacts with Fanconi anemia protein complexes to promote DNA damage response
Source: Oncotarget. 2015 Nov 28;7(2):1912–26. doi: 10.18632/oncotarget.6422 (PMC4811506; doi:10.18632/oncotarget.6422)
Supplement: Supplementary file 1 [file oncotarget-07-1912-s001.pdf]

Forkhead transcription factor FoxF1 interacts with Fanconi anemia protein complexes to promote DNA damage response

Supplementary Materials

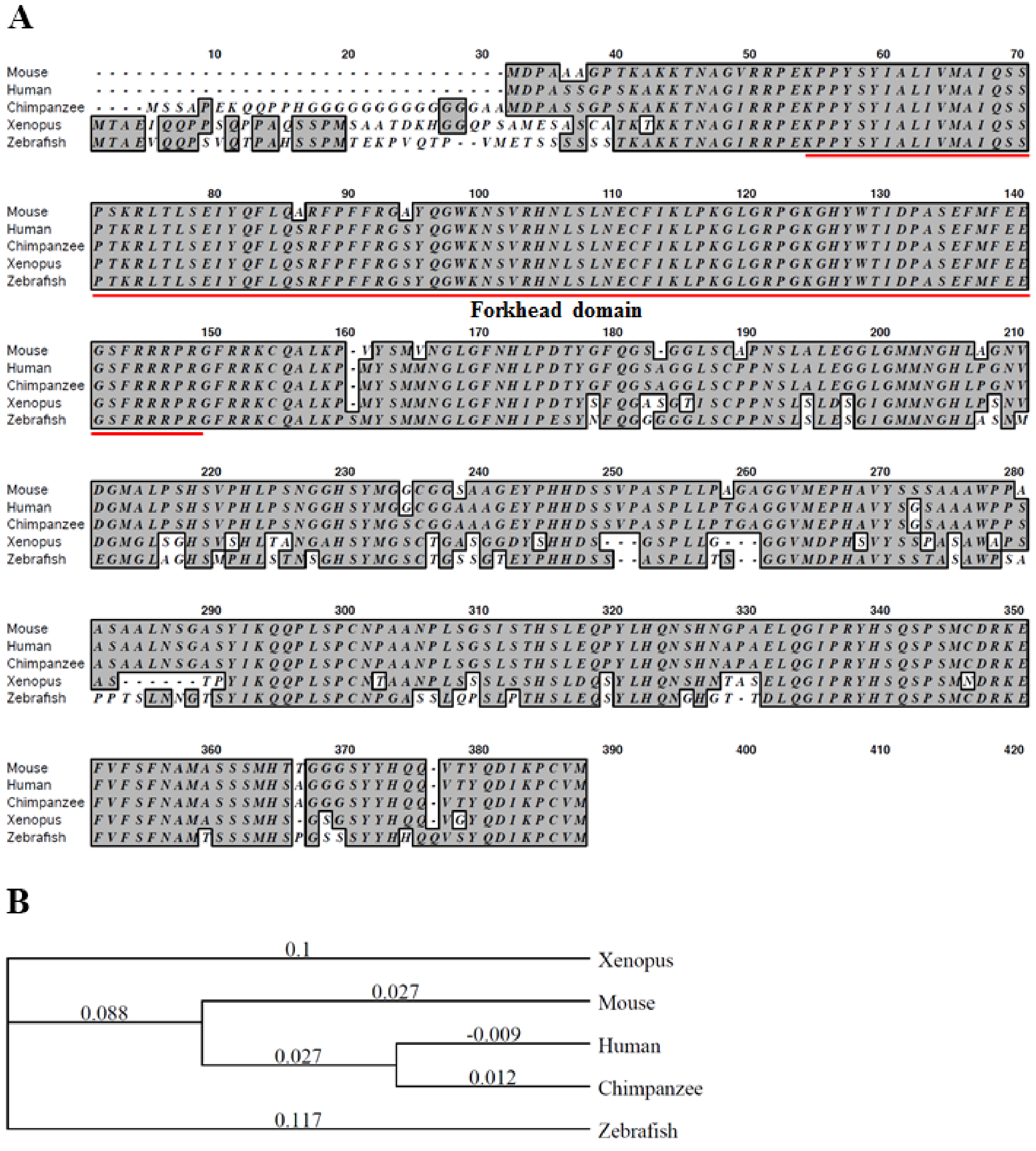

Supplementary Figure S1: Sequence homology in FoxF1 gene. (**A**) Comparison of amino acid sequence of FoxF1 from Mus musculus (Accession number NP_034556) with FoxF1proteins from Homo sapiens (Accession number AAC50399), Pan troglodytes (chimpanzee) (Accession number xp_523449), Xenopus laevis (Accession number Q9W707) and Danio rerio (zebrafish) (Accession number A1L1S5). Multiple alignments were performed using CLUSTALW program (Mac vector version 9.5.2). Identical amino acids of each protein are indicated with black boxes. Forkhead domain is shown with red line. (**B**) Phylogenetic tree was determined using CLUSTALW program (Mac vector version 9.5.2) based on the multiple alignment of amino-acid sequences of FoxF1 proteins from different species. A value of 0.1 corresponds to a difference of 10% between amino acid sequences.

Supplementary Table S1: List of antibodies used for western blot, IP and immunostaining

1.	FANCM: SC-98710 (Santa Cruz Biotechnology)
2.	FANCA: SC-18664 (Santa Cruz Biotechnology)
3.	FAAP100: GTX116871 (GeneTex, Inc.)
4.	FoxA2: WRAB-1200 (Seven Hills Bioreagents)
5.	FoxA3: SC-5361 (Santa Cruz Biotechnology)
6.	FoxE1: SC-16391 (Santa Cruz Biotechnology)
7.	FoxF1: AF4798(R&D Systems)
8.	FoxJ1: WMAB-319 (Clone 3-19) (Seven Hills Bioreagents)
9.	FoxM1: SC-502 (Santa Cruz Biotechnology)
10.	FAAP20: GTX51439 (GeneTex, Inc.)
11.	MHF1: OAAB02336 (Aviva Systems Biology, Corp.)
12.	MHF2: ARP52780 (Aviva Systems Biology, Corp.)
13.	FANCI: A300-212A (Bethyl Laboratories, Inc)
14.	FANCD2: ab2187 (Abcam)
15.	FANCL(B-11): SC-137067 (Santa Cruz Biotechnology)
16.	FAAP24: SC-167769 (Santa Cruz Biotechnology)
17.	Lamin A/C: SC-20681 (Santa Cruz Biotechnology)
18.	Actin: SC-1615 (Santa Cruz Biotechnology)
19.	α-Tubulin: ab125267 (Abcam)
20.	α-Flag (clone M2): (F-1804) (Sigma)
